# Photosynthetic dependence and filament production in physical bacterial–Symbiodiniaceae interactions

**DOI:** 10.1093/ismeco/ycaf070

**Published:** 2025-04-25

**Authors:** Gavin C McLaren, Morgan V Farrell, Nicholas J Shikuma, Cawa Tran

**Affiliations:** Department of Biology, University of San Diego, San Diego, CA 92110, United States; Department of Biology, San Diego State University, San Diego, CA 92182, United States; Department of Biology, San Diego State University, San Diego, CA 92182, United States; Department of Biology, University of San Diego, San Diego, CA 92110, United States

**Keywords:** algae, dinoflagellate, cnidarian, microbiome, symbiosis, holobiont, microscopy

## Abstract

The cnidarian microbiome consists of a wide variety of beneficial microbes that play vital roles in maintaining and fortifying host health. Photosynthesis from symbiotic dinoflagellates (in the family Symbiodiniaceae) is crucial for their symbiosis establishment with the cnidarian host. Although more is known regarding interactions between the host and its associated bacteria and dinoflagellates, there has been little investigation into the relationship between the two microbes themselves and whether photosynthesis plays a role. Through two different methods of photosynthetic inhibition of dinoflagellates (incubation in the dark or pre-treatment with a photosystem II inhibitor), we investigated how pathogenic versus beneficial bacteria physically interact with three Symbiodiniaceae strains (symbiotic and free-living). The beneficial bacterium *Tritonibacter mobilis* appears to interact with photosynthesizing algae only. In the absence of photosynthesis, little to no physical interactions were observed between Symbiodiniaceae and *T. mobilis*. Bacterial congregation around individual dinoflagellate cells was significantly lower when photosynthesis was impaired, suggesting photosynthesis is a key facilitator of interactions between *T. mobilis* and all three Symbiodiniaceae strains. We also investigated whether photosynthesis affects interactions between Symbiodiniaceae and the pathogen *Vibrio alginolyticus*. Although no discernable impacts of photosynthetic inhibition were observed with the pathogen, scanning electron microscopy uncovered various mechanisms of interaction between Symbiodiniaceae and both bacteria, one of which includes the production of filaments not previously described. Overall, our research highlights the importance of photosynthesis in initiating interactions between bacteria and both free-living and symbiotic dinoflagellates, and opens a door to new questions regarding cell-surface interactions among individual microbes.

## Introduction

Dinoflagellates and bacteria are an integral part of ocean ecosystems in their free-living forms [1], but they also independently establish important relationships with a wide variety of marine animals as symbiotic partners. Symbiodiniaceae, a specific family of dinoflagellates, forms a mutualism with different cnidarian hosts, such as corals, sea anemones, and jellyfish, providing them with photosynthetic products in exchange for inorganic nutrients and protection from grazing [2]. Bacteria are a major component of the cnidarian microbiome, with a range of functions from nutrient cycling to pathogen defense for the host [3]. However, the possible interactions between dinoflagellates and bacteria within their hosts remain largely unexplored [4]. The predominant hypothesis, at least among free-living representatives, is that dinoflagellates recruit bacteria via production of dissolved organic matter and may, in turn, benefit from essential vitamins and nutrients (e.g. bioavailable nitrogen and phosphorus) provided by bacteria [1]. There is limited knowledge of whether photosynthesis has a role in establishing Symbiodiniaceae-bacterial interactions.

Whether in symbiotic or free-living forms, these microbes will become increasingly susceptible to the impacts of global climate change. High ocean temperatures result in the loss of photosynthetic capacity in symbiotic algae, as established across many previous studies [5–10]. An impairment of photosynthesis, characterized by the loss of chlorophyll pigmentation and waning photochemical output from chloroplasts [5, 11, 12], induces host stress in the form of coral bleaching [5, 13, 14]. It is unknown whether photosynthetic impairment may also impact algal interactions with any surrounding bacteria, with or without the host.

Symbiodiniaceae are diverse in their morphology and life history [15], which may potentially influence their interactions with bacteria. Thus, three species of Symbiodiniaceae with very different characteristics—*Breviolum minutum*, *Effrenium voratum*, and *Symbiodinium pilosum*—may contribute differently to host-microbe and microbe-microbe interactions. *B. minutum* is smaller in size relative to other Symbiodiniaceae and compatible in establishing symbiosis with corals and sea anemones [12, 16–18]. *E. voratum* is a naturally non-symbiotic, or free-living, Symbiodiniaceae species [19], and one of the largest in volume compared to any of its Symbiodiniaceae relatives [2]. *S. pilosum* was first isolated from the green button polyp, *Zoanthus sociatus*, but is not known to be taken up by corals or sea anemones [12, 18, 20–23]. These three species of Symbiodiniaceae also vary in surface morphology. Scanning electron microscopy (SEM) and light microscopy indicate that, without the exogenous presence of glucose, the natural morphology of *B. minutum* is rough and complex [24]. Cells of *E. voratum* are larger in size (relative to cells of *B. minutum* and *S. pilosum*) and many maintain a motile state, composed of thecal plates and transverse flagella for movement [19]. *S. pilosum* is unique from *B. minutum* and *E. voratum* in having a tufted or pilose exterior via light-microscopy analysis [21].

Although there has been extensive research regarding bacteria and Symbiodiniaceae independently benefitting their animal host, there has been limited research (until recently) on any possible interactions between the two microbes [4, 25], and their implications for host health. *Tritonibacter* (formerly *Ruegeria* [26]) is a widely abundant bacterial symbiont of corals, with 356 sequences (based on the 16S ribosomal ribonucleic acid gene) spanning across 36 different species of corals [27]. *Tritonibacter* and other Paracoccaceae (formerly Rhodobacteraceae) are well associated with the sea anemone *Exaiptasia diaphana* (commonly referred as and hereafter “Aiptasia”) [28]. Rhodobacterales (the order in which *Tritonibacter* resides), in fact, is even known to colonize the early life stages of corals [29]. In situ experiments reveal that the abundance of *Tritonibacter* members increases in healthy corals when treated with probiotics [30]. Additionally, it has been proposed that many symbiotic *Tritonibacter* species may provide beneficial effects to host health, such as inhibition of opportunistic pathogen growth [31], protection of microbiome diversity [32], and degradation of pollutants in coral mucus [33]. New evidence [34] presents significant differences in animal-protein content between symbiotic (with Symbiodiniaceae) and aposymbiotic (without Symbiodiniaceae) Aiptasia when inoculated with *Tritonibacter mobilis*, which suggests a possible interaction between the two microbes. A related bacterium, *Ruegeria pomeroyi*, forms mutualistic relationships with another photosynthetic microbe, a diatom [35]. This begs the question of whether its relative, *T. mobilis,* could have similar mutualistic interactions with the photosynthetic microbe in its host environment, the Symbiodiniaceae.

Marine bacteria are physiologically and functionally diverse. Beneficial bacteria help fortify the health of their animal host, whereas pathogens compromise them. Despite *Vibrio* being a dominant genus associated with corals [27, 36], many key *Vibrio* spp*.* are known pathogens of marine animals, associated with diseases and coral bleaching [28, 37–41]. Specifically, *Vibrio alginolyticus* is associated with white syndrome in *Porites andrewsi* [39, 42] and yellow band disease [38], which are serious coral diseases tied to bleaching. *Vibrio shiloi* causes bleaching and lysis of Symbiodiniaceae subsequently after adhesion to surface β-D-galactopyranoside of host tissues in the coral *Oculina patagonica* [43–46]. Such findings promote inquiry into possible physical interactions directly between pathogenic *Vibrio* spp*.* and Symbiodiniaceae as well.

The main goal of this research is to understand the intercellular dynamics of Symbiodiniaceae and bacteria upon initiation of physical contact. There has been limited research done to determine what facilitates this initial attraction between the two microorganisms. One possibility is algal photosynthesis. We hypothesize that inhibiting photosynthesis within algal cells may diminish their physical interactions with two different bacterial species, and tested this *ex hospite* with high-resolution fluorescence and SEM.

## Materials and methods

### Fluorescence-tagging of *Tritonibacter mobilis* and *Vibrio alginolyticus*

*Tritonibacter mobilis* AipH2 and *Vibrio alginolyticus* AipCC7 strains were originally isolated from the H2 and CC7 clonal lines of Aiptasia, respectively. To enable their visualization for fluorescence microscopy, both strains were fluorescently tagged with pMMK819, which is a mini Tn7 transposon integration element that inserts downstream of the glucosamine-6-phosphate riboswitch (*glmS*) gene. The Tn7 insertion contains a constitutive promoter (*CP25*) driving a green fluorescent protein (*gfp*). The bacterial strain MFD-λpir containing pMMK819 was grown on Luria–Bertani (LB Miller, BD Difco) with 50 μg ml^−1^ of kanamycin, 10 μg ml^−1^ of gentamycin, and 0.03 mM of diaminopimelic acid (DAP). *E. coli* S17–1 containing the helper plasmid pUX-BF13, which carries the transposase, was grown on LB with 100 μg ml^−1^ ampicillin and 0.3 mM DAP. *T. mobilis* AipH2 and *V. alginolyticus* AipCC7 were grown in marine broth (MB, BD Difco). A triparental mating was performed with a 1:1:1 mating ratio of MFD-λpir pMMK819, S17–1 pUX-BF13, and marine strains. *E. coli* donor strains were auxotrophic MFD-λ*pir* requiring DAP for growth**.** Mating was performed as previously described [47]. Briefly, three colonies of marine strains were inoculated and grown overnight in liquid culture at 25°C. A single colony of each *E. coli* strain was inoculated in liquid culture with appropriate antibiotics and grown overnight at 37°C. Cultures were normalized to 1:1:1 ratio based on optical density at 600 nm (OD_600_), then spun down at 4000 × g for 10 min. The supernatant was removed, and cultures were resuspended in 150 μl of MB**.** Negative controls were spotted (1, 50 μl spot) onto marine agar. Mating resuspensions were mixed and then spotted (4, 50 μl spots) onto marine agar and incubated overnight at 25°C. Spots were scraped up and resuspended in MB, washed 2× and then 100 μl were plated on marine agar with gentamycin 200 μg ml^−1^.

### Algal strains and growth conditions

SSB01 (*B. minutum*; ITS2 Clade B1), SSE01 (*E. voratum*; ITS2 Clade E), and SSA03 (*S. pilosum*; ITS2 Clade A2) are axenic strains of Symbiodiniaceae developed by the Pringle and Grossman Labs (Stanford, CA) by depleting their nascent bacterial communities [22]. Algal stocks were grown in IMK (sterile seawater containing 0.25 g L^−1^ Daigo’s IMK powder; FujiFilm Wako Chemicals) for 14 days at 27°C under a 12 h: 12 h light–dark cycle using white LEDs at an irradiance of 25 μmol photons m^−2^ s^−1^ of photosynthetically active radiation as measured with a GMSW-SS quantum meter (Apogee). Symbiodiniaceae cultures were routinely inspected visually and plated onto marine agar to verify axenic conditions, especially before experiments were conducted. Algal densities of all three strains were determined via hemocytometer on an automated cell counter (Corning, Tewksbury, MA). Autofluorescence from their natural chlorophyll pigments provided ease of visualization under fluorescence microscopy.

### Pretreatment of algae for photosynthetic inhibition

3-(3,4-dichlorophenyl)-1,1-dimethylurea (DCMU) is a photosystem II inhibitor that prevents glucose production from algae [12, 48–50]. Aliquots of 1 ml of each Symbiodiniaceae strain were pretreated under the following conditions in 1.5-ml microcentrifuge tubes (Fig. S1): (i) 12 h light:12 h dark, (ii) continuous dark, and (iii) 10 μM DCMU. All tubes were incubated at 27°C under a 12 h light:12 h dark cycle for 4 days. The “continuous dark” condition was the result of wrapping the tubes in foil to block out light. Three aliquots of each condition (12 h light:12 h dark, continuous dark, and 10 μM DCMU) for each algal strain (SSB01, SSA03, and SSE01) were established.

### Preparation of bacteria and algae for inoculation

*T. mobilis* AipH2-GFP and *V. alginolyticus* AipCC7-GFP were grown in MB for 24 h at 27°C, shaking at 200 rpm. OD_600_ for overnight cultures was measured with a Thermofisher Scientific Spectronic 200E, resulting in ranges of 0.9–1.3 for both bacterial strains. Cultures were centrifuged at 14.5 krpm for 15 min to pellet bacterial cells. The MB supernatant was then replaced with sterile seawater (SSW), adjusting to OD_600_ = 0.2.

Pretreated algae (see above) were spun down via centrifuge at 14.5 krpm for 15 min to pellet cells and replace IMK media with SSW to obtain a density of 4.0 × 10^5^ cells ml^−1^. Each combination of Symbiodiniaceae and bacterial strain was mixed in a 12-well plate in a 1:1 ratio per well, resulting in a final algal density of 2.0 × 10^5^ cells ml^−1^ and OD_600_ = 0.1 for *T. mobilis* AipH2-GFP and *V. alginolyticus* AipCC7-GFP. These algal and bacterial densities were within the ranges separately tested with the sea anemone Aiptasia in previous studies [12, 34], and combining these algae and bacteria together in a 1:1 ratio in this present *ex hospite* study was a logical starting point.

### Fluorescence microscopy and data analysis

All algal–bacterial combinations were analyzed approximately 15 min post-inoculation, the shortest time frame needed to prepare cells for microscopy that still enabled examination of critical interactions upon initial contact between algae and bacteria. Each group was mixed via pipetting up and down to dislodge any algal cells from the bottom of wells for collection. Algal–bacterial mixtures mounted on glass slides were imaged using an Olympus BX51 fluorescence compound microscope at 400× magnification under DIC, FITC, and TRITC channels. FITC and TRITC images were taken under 750 and 100 ms exposure, respectively. Algal clusters larger than 4 cells were omitted from data collection and analysis to focus on bacteria and their interactions with individual algal cells. To quantify the degree of physical interaction between bacterial cells and the algal-cell surface, bacterial fluorescence intensity around the immediate area of each Symbiodiniaceae cell was measured with Image J (version 1.54f) [51]. Regions of interest were measured with rectangles standardized for each individual algal strain, due to variable cell sizes (in which SSE01 and SSA03 are larger than SSB01): SSB01 (w = 59, h = 56), SSE01 (w = 75, h = 75), SSA03 (w = 75, h = 75). For examinations of *T. mobilis* AipH2-GFP interacting with algae, sample sizes ranged from 22–39 cells of SSB01, 31–40 cells of SSE01, and 40–50 cells of SSA03, across three independent trials of the experiment. For examinations of *V. alginolyticus* AipCC7-GFP interacting with algae, sample sizes ranged from 29–34 cells of SSB01, 34–37 cells of SSE01, and 19–34 cells of SSA03, across three independent trials of the experiment. A one-way analysis of variance (ANOVA) followed by a post-hoc Tukey’s test was conducted for each algal–bacterial combination for statistical analysis.

### Scanning electron microscopy

Inoculations of bacteria and algae (that had been growing under 12 h light:12 h dark) were performed exactly as described above. Each algal–bacterial mixture was transferred onto a poly-L-lysine-coated 12-mm coverslip (Electron Microscopy Sciences, Hatfield, PA) and allotted approximately 15 min to settle before fixing with 2% glutaraldehyde and 4% paraformaldehyde in 0.1 M Na-cacodylate buffer (pH 7.3). Fixed samples were then treated in 1% aqueous OsO_4_ for 1 h. Afterward, samples underwent an increasing serial dilution of ethanol (50%, 70%, 90%, and 100%). Samples were subsequently dehydrated using a SAMDRI PVT-3 D critical point dryer. Glass coverslips were mounted onto a Hitachi M4 aluminum specimen mount (15 × 6 mm, Ted Pella, Inc., Redding, CA) and coated with Au/Pa with an Emitech K550X sputter coater. Coated samples were then imaged with a Hitachi 3400 N scanning electron microscope. Brightness and contrast of images were adjusted in Image J for clarity.

### Genomic characterization of bacterial strains

To verify the classification of bacterial strains and examine key genes potentially involved in metabolizing photosynthetic products, the genomes of *T. mobilis* AipH2 and *V. alginolyticus* AipCC7 were sequenced, assembled, and annotated according to a previously published method [52] (see Supplementary Materials and Methods, Figs S2 and S3). ModelSEED v2.6.1 [53] analysis was performed on bacterial strains *T. mobilis* AipH2 and *V. alginolyticus* AipCC7 to assess potential pathways involved in glucose and glycerol metabolism (Fig. S4).

### Physiological characterization of bacterial strains

For phenotypic validation of GFP-tagged strains to assess possible impacts of GFP insertion, we compared the growth of *T. mobilis* AipH2, *T. mobilis* AipH2-GFP, *V. alginolyticus* AipCC7, and *V. alginolyticus* AipCC7-GFP at various temperatures (see Supplementary Materials and Methods, Fig. S5). For assessment of physiological responses to different carbon sources relevant to the photosynthetic pathway, substrate preferences of *T. mobilis* AipH2, *T. mobilis* AipH2-GFP, *V. alginolyticus* AipCC7, and *V. alginolyticus* AipCC7-GFP were examined (Fig. S6). All four strains were grown on agar plates of M9 minimal media (BD Difco) supplemented with 0.4% glucose, 0.4% glycerol, or 0.4% casein hydrolysate as carbon sources relevant to host-microbe symbioses. Glucose and glycerol are the major metabolites translocated between cnidarians and Symbiodiniaceae [50, 54], whereas casein hydrolysate supports the growth of some Symbiodiniaceae in culture [22]. Two sets of M9 minimal media (with these various supplements) were made, one in deionized water and the other in seawater to determine differences in their ability to support growth of these marine bacteria. The latter produced some particulates when M9 was combined with seawater. All four bacterial strains were also simultaneously grown on marine-agar plates for comparison. All plates were incubated at 27°C for 24–72 h to assess growth of colonies for each strain, as different media types required different incubation times for bacteria to grow.

## Results

### Inhibition of photosynthesis decreases localization of *Tritonibacter mobilis* to algae

To investigate the influence of photosynthesis on interactions between the beneficial bacterium, *T. mobilis*, and dinoflagellates, we turned to fluorescence microscopy to examine physical intercellular interactions within initial exposure of each other. Algal strains SSB01 (*B. minutum)*, SSE01 (*E. voratum)*, and SSA03 (*S. pilosum)* were examined under the microscope shortly after inoculation (approximately 15 min) with *T. mobilis* AipH2-GFP, chromosomally tagged with pMMK819 (mini Tn7 transposon) inserted downstream of the *glmS* gene, for visualization using fluorescence microscopy.

**Figure 1:**
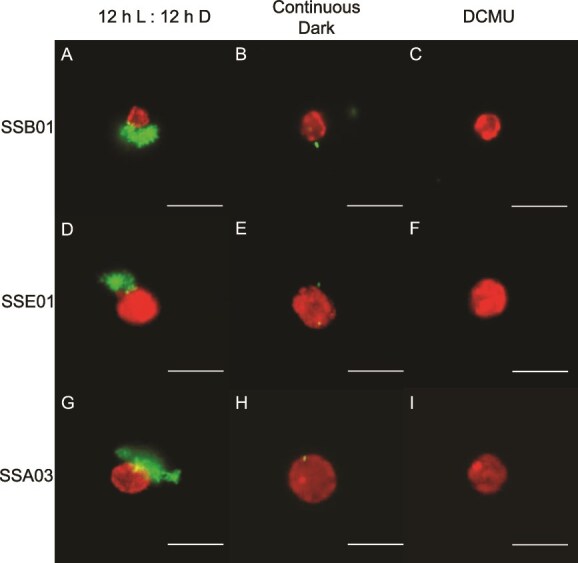
Photosynthetic inhibition of Symbiodiniaceae reduces the localization of *Tritonibacter mobilis* (bacteria) to their algal-cell surfaces*.* Symbiodiniaceae strains SSB01 (A, B, C), SSE01 (D, E, F), and SSA03 (G, H, I) were pretreated for four days in a 12 h light:12 h dark cycle without DCMU (control; A, D, G), continuous dark cycle (B, E, H), or 12 h light:12 h dark cycle with 10 μM DCMU (C, F, I). IMK media was then replaced with sterile seawater (SSW) and GFP-tagged *T. mobilis* in SSW was introduced. Final concentrations of algae and bacteria in SSW were 2.0 × 10^5^ cells ml^−1^ and OD_600_ = 0.1, respectively. Fluorescence imaging took place approximately 15 min after inoculation*.* Red, chlorophyll fluorescence of algae; green, recombinant GFP fluorescence of tagged *T. mobilis.* Scale bars represent 10 μm.

**Figure 2:**
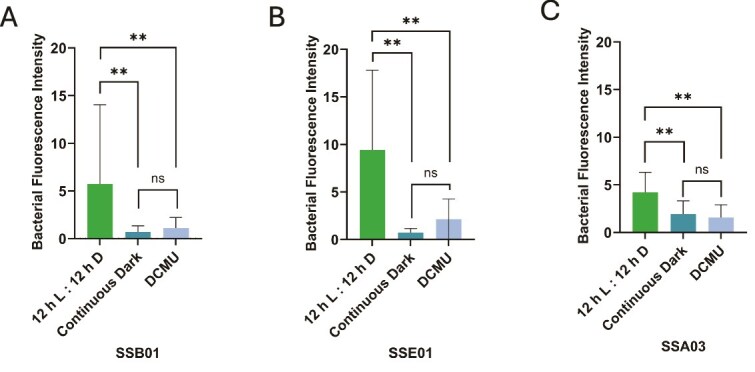
Inhibition of algal photosynthesis decreases *Tritonibacter mobilis* (bacteria) localization to Symbiodiniaceae. Algal strains (A) SSB01, (B) SSE01, and (C) SSA03 underwent the same three pretreatments: 12 h light:12 h dark cycle without DCMU (control), continuous dark cycle, or 12 h light:12 h dark cycle with 10 μM DCMU. Bacterial fluorescence (FITC channel) was measured in ImageJ. Sample sizes ranged from 29–34 algal cells for SSB01, 34–37 algal cells for SSE01, and 19–34 algal cells for SSA03, based on three independent trials of the experiment. Bars represent average bacterial fluorescence intensity + SD. A one-way ANOVA followed by a post-hoc Tukey’s test show significant differences where indicated. ^**^P = .001. ns = not significant.

Upon imaging the three algal strains under normal conditions (12 h light:12 h dark, no DCMU), *T. mobilis* AipH2-GFP was observed to localize directly around Symbiodiniaceae (red) in star-shaped clusters of varying sizes (Fig. 1A, D, and G). Algae were also subjected to two methods of photosynthesis suppression: continuous dark and 10 μM DCMU. Both methods of photosynthesis inhibition revealed stark decreases in *T. mobilis* AipH2-GFP around algal cells of all three Symbiodiniaceae strains (Fig. 1B, C, E, F, H, and I). Bacterial fluorescence intensity surrounding SSB01 dinoflagellates under normal conditions, when quantified with ImageJ, was over eight and four times greater than under continuous dark and DCMU, respectively [Fig. 2A; F(2, 96) = 11.53, *P* = .001]. When inoculated with the free-living dinoflagellate, SSE01, normal conditions produced an average fluorescence more than 12 and seven times greater than continuous dark and DCMU, respectively [Fig. 2B; F(2, 104) = 28.10, *P* = .001]. Under normal conditions with SSA03, *T. mobilis* AipH2-GFP displayed immediate peripheral fluorescence intensities over two times greater than both photosynthesis-inhibition groups [Fig. 2C; F(2, 76) = 18.46, *P* = .001]. There were no significant differences between continuous dark and DCMU when *T. mobilis* AipH2-GFP was exposed to SSB01 (*P* = .899), SSE01 (*P* = .482), and SSA03 (*P* = .656) strains (Fig. 2).

### Attachment of *Tritonibacter mobilis* to different algal strains

**Figure 3:**
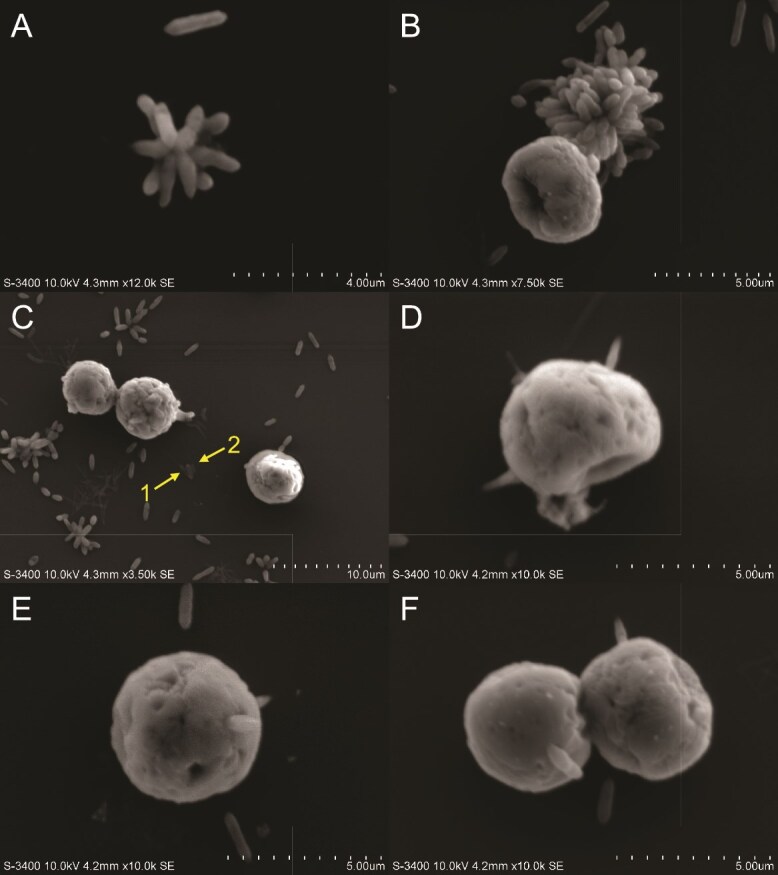
Scanning electron micrographs (SEM) of *Tritonibacter mobilis* (bacteria) physically interacting with Symbiodiniaceae strain SSB01. Algal cells were grown in IMK medium in a 12 h light:12 h dark cycle. Media was replaced with sterile seawater (SSW) and inoculated with *T. mobilis* in SSW. Final concentrations of algae and bacteria in SSW were 2.0 × 10^5^ cells ml^−1^ and OD_600_ = 0.1, respectively. Algal–bacterial mixture was fixed approximately 15 min after inoculation for SEM (see materials and methods). (A, B) Star-shaped clusters of *T. mobilis*. (C–F) Specific cell–cell interactions between the polar ends of individual *T. mobilis* rods and SSB01. Leaf-shaped pads (arrows) at the ends of filamentous appendages protrude from the algal surface and interact with a bacterial rod.

To visualize the physical interactions between bacteria and Symbiodiniaceae in greater detail, we performed SEM. These electron micrographs allowed us to make a deeper analysis and examine the textures of different cell surfaces along with other features not detected by fluorescence microscopy. SEM images of *T. mobilis* AipH2-GFP with SSB01 (Fig. 3) reveal localization of bacteria both directly on and within the immediate proximity of the algal-cell surface. *T. mobilis,* as previously described [55], forms star-shaped clusters in sizes that vary quite extensively (Fig. 3A and B). Within these clusters, the point of contact between the individual rods is shown to be only their polar ends. Non-clustering interactions between *T. mobilis* and SSB01 involve multiple rods individually attached to the algal surface by their polar ends (Fig. 3C–F).

**Figure 4:**
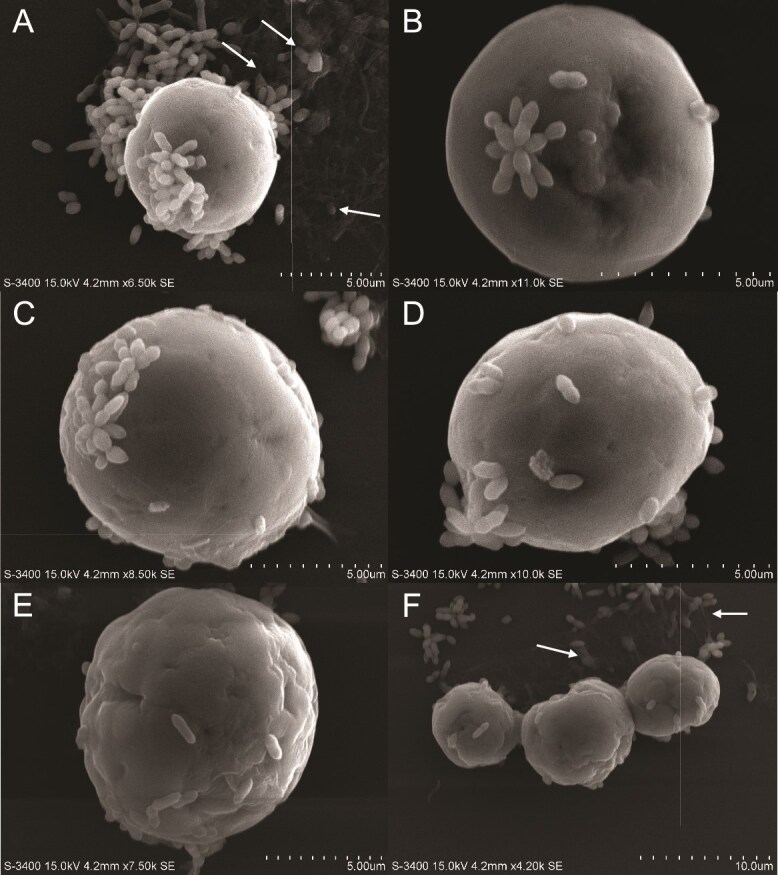
SEM of *Tritonibacter mobilis* (bacteria) physically interacting with Symbiodiniaceae strain SSE01. Algal cells were grown in IMK medium in a 12 h light:12 h dark cycle. Media was replaced with sterile seawater (SSW) and inoculated with *T. mobilis* in SSW. Final concentrations of algae and bacteria in SSW were 2.0 × 10^5^ cells ml^−1^ and OD_600_ = 0.1, respectively. Algal–bacterial mixture was fixed approximately 15 min after inoculation for SEM (see materials and methods). (A–E) Individual SSE01 cells interacting with *T. mobilis*. (F) Zoomed-out view of an SSE01 cluster with bacteria and surrounding extracellular matrix (ECM). ECM is labeled with arrows.

Subtle yet key differences in intercellular interactions exist between SSE01 and *T. mobilis* (Fig. 4). The surface texture of SSE01 is uneven and wrinkled, like SSB01, but these features are slightly shallower and smoother. In contrast to SSB01, in which *T. mobilis* rods were observed attaching individually to the dinoflagellate, star-shaped *T. mobilis* clusters maintain their astral configuration while fanned and spread out (as if pasted) onto the SSE01 cell (Fig. 4A–D), ensuring maximum surface-area attachment. In addition to these clusters, individual rods establish both polar-end and lateral connections with the algal-cell surface (Fig. 4D–F).

**Figure 5:**
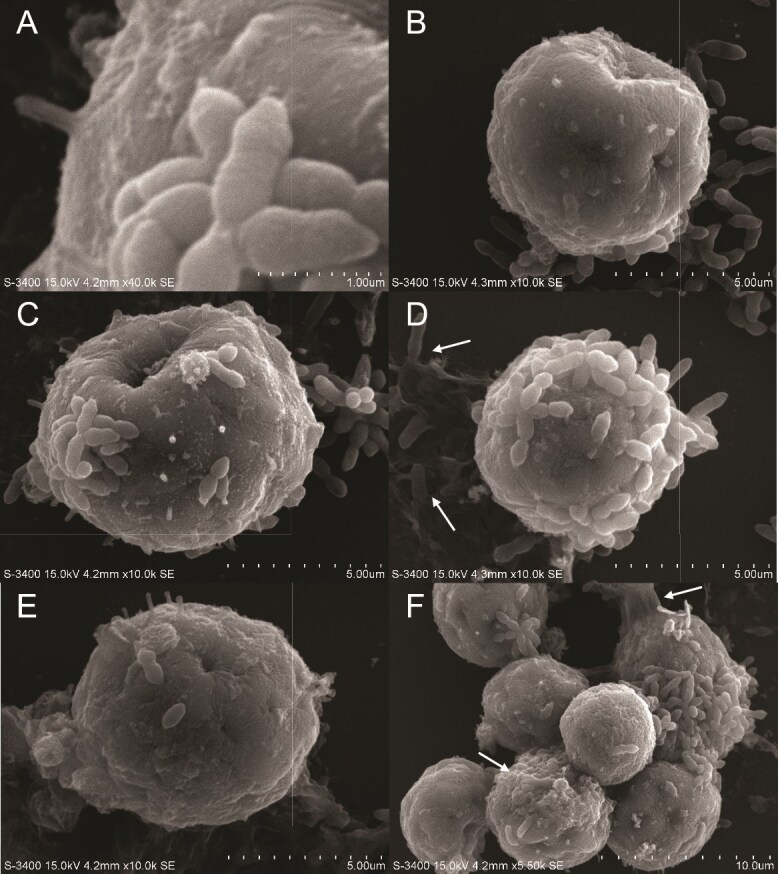
SEM of *Tritonibacter mobilis* (bacteria) physically interacting with Symbiodiniaceae strain SSA03. Algal cells were grown in IMK medium in a 12 h light:12 h dark cycle. Media was replaced with sterile seawater (SSW) and inoculated with *T. mobilis* in SSW. Final concentrations of algae and bacteria in SSW were 2.0 × 10^5^ cells ml^−1^ and OD_600_ = 0.1, respectively. Algal–bacterial mixture was fixed approximately 15 min after inoculation for SEM (see materials and methods). (A) Magnified image of SSA03 tuft on cell surface next to and *T. mobilis* cluster. (B–E) Individual SSE01 cells interacting with *T. mobilis*. (F) Zoomed-out view of an SSE01 cluster with bacteria and ECM. ECM is labeled with arrows.

In contrast to SSB01 and SSE01, SSA03 has a rougher surface texture, uniquely covered in tuft-like extensions (Fig. 5A and B) that are reminiscent of the pilose surface described by Trench and Blank [20] and LaJeunesse [21] of this species. This tuft (Fig. 5A) measures to be around 500 nm, appearing to be completely cylindrical with a round tip. In terms of bacterial attachment to this dinoflagellate, *T. mobilis* interacts with SSA03 in a remarkably similar manner to SSE01. There were many cases in which the bacteria attached to the algae as both star-shaped clusters (Fig. 5C and D) and individual rods (Fig. 5E and F). Despite SSA03 having the unique characteristic of a pilose surface, this does not seem to influence any distinct interactions with the bacteria. Overall, *T. mobilis* both individually and in clusters attach to the surfaces of different Symbiodiniaceae species in similar orientations.

### Localization of *Vibrio alginolyticus* to algae unchanged by photosynthetic inhibition

**Figure 6:**
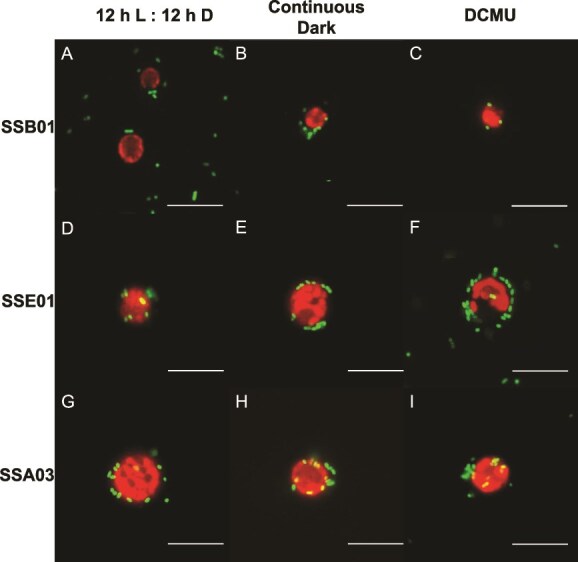
Photosynthetic inhibition of Symbiodiniaceae does not impact their interactions with *Vibrio alginolyticus* (bacteria)*.* Symbiodiniaceae strains SSB01 (A, B, C), SSE01 (D, E, F), and SSA03 (G, H, I) were pretreated for four days in a 12 h light:12 h dark cycle without DCMU (control; A, D, G), continuous dark cycle (B, E, H), or 12 h light:12 h dark cycle with 10 μM DCMU (C, F, I). IMK media was then replaced with sterile seawater (SSW) and GFP-tagged *V. alginolyticus* in SSW was introduced. Final concentrations of algae and bacteria in SSW were 2.0 × 10^5^ cells ml^−1^ and OD_600_ = 0.1, respectively. Fluorescence imaging took place approximately 15 min after inoculation*.* Red, chlorophyll fluorescence of algae; green, recombinant GFP fluorescence of tagged *V. alginolyticus.* Scale bars represent 10 μm.

**Figure 7:**
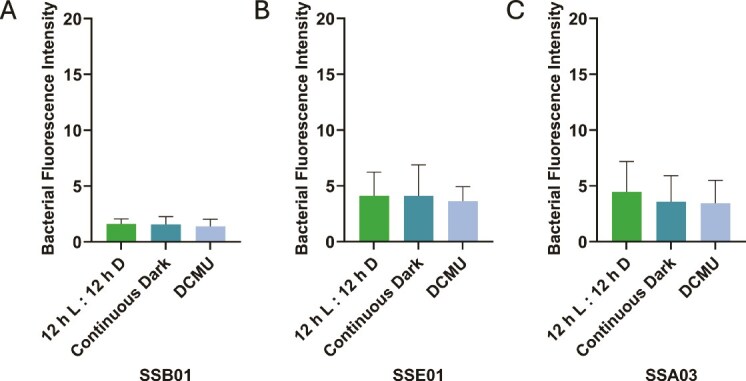
Localization of *Vibrio alginolyticus* (bacteria) to Symbiodiniaceae is unchanged by photosynthetic inhibition. Algal strains (A) SSB01, (B) SSE01, and (C) SSA03 underwent the same three pretreatments: 12 h light:12 h dark cycle without DCMU (control), continuous dark cycle, or 12 h light:12 h dark cycle with 10 μM DCMU. Bacterial fluorescence (FITC channel) was measured in ImageJ. Sample sizes ranged from 22–39 algal cells for SSB01, 31–40 algal cells for SSE01, and 40–50 algal cells for SSA03, based on three independent trials of the experiment. Bars represent average bacterial fluorescence intensity + SD. A one-way ANOVA indicated no significant differences.

To test whether beneficial and pathogenic bacteria differ in their interaction with Symbiodiniaceae in the presence and absence of light or a photosynthesis inhibitor, we subjected the same three algal strains to the pathogen *V. alginolyticus* AipCC7-GFP, chromosomally tagged with pMMK819 (mini Tn7 transposon) inserted downstream of the *glmS* gene, under the same experimental conditions. Under normal conditions, multiple *V. alginolyticus* AipCC7-GFP rods congregated directly around all dinoflagellate strains (Fig. 6A, D, G). Both photosynthesis-inhibition methods also resulted in bacterial congregation around the algae (Fig. 6B, C, E, F, H, and I). Interactions of *V. alginolyticus* AipCC7-GFP with Symbiodiniaceae were in stark contrast to those of *T. mobilis.* Cells of *V. alginolyticus* individually establish themselves around the periphery of the algal cells rather than clusters of any size. Across all photosynthesis conditions, no significant differences in bacterial fluorescence intensity were observed in SSB01 [*F*(2, 92) = 1.11, *P* = .333], SSE01 [*F*(2, 100) = 0.84, *P* = .435], nor SSA03 [*F*(2, 136) = 2.24, *P* = .111; Fig. 7].

**Figure 8:**
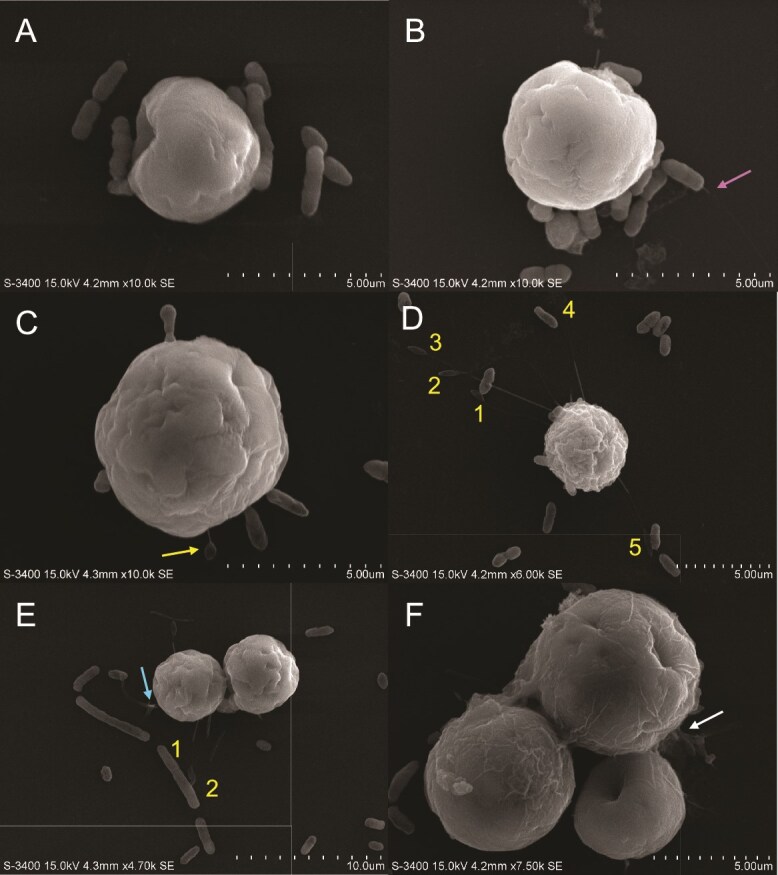
SEM of *Vibrio alginolyticus* (bacteria) physically interacting with Symbiodiniaceae strain SSB01. Algal cells were grown in IMK medium in a 12 h light:12 h dark cycle. Media was replaced with sterile seawater (SSW) and inoculated with *T. mobilis* in SSW. Final concentrations of algae and bacteria in SSW were 2.0 × 10^5^ cells ml^−1^ and OD_600_ = 0.1, respectively. Algal–bacterial mixture was fixed approximately 15 min after inoculation for SEM (see materials and methods). (A, B) individual SSB01 cells interacting with *V. alginolyticus* with a bacterial flagellum present (pink arrow). (C) Individual SSB01 cell interacting with *V. alginolyticus.* SSB01 exhibits a small protruding filament attached to the poly-L-lysine-coated glass substratum (arrow). (D) SSB01 cell with multiple protruding filaments (numbers) which interact with either a bacterium or the glass substratum. (E) SSB01 cell with two protruding filaments (numbers) and two flagella branching from a peduncle (arrow). (F) Zoomed-out view of an SSB01 cluster covered in ECM (arrow) with surrounding bacteria.

Adhesion of *V. alginolyticus* AipCC7-GFP to SSB01, as determined by SEM, differs greatly from *T. mobilis.* Instead of attaching to the algal surface by their polar ends like *T. mobilis* (Fig. 3), *V. alginolyticus* laterally adheres to the algal surface (Fig. 8A–D). Some appear partially embedded into the algal surface (Fig. 8D). Unlike *T. mobilis*, the pathogen does not form star-shaped clusters with each other, but rather chains instead (Fig. 8E).

### Filaments form during algal interactions with bacteria and substratum

When combined with either bacterial species, SSB01 extends unknown filaments from its cell body. Very thin and tubular in the middle, ending with a leaf-shaped pad, these filaments make physical connections with either the poly-L-lysine-coated glass substratum (Fig. 8C and D) or the bacteria (Fig. 8D and E) in the environment. The leaf-shaped pads are unlikely to be bacterial rods, as they are half the size of an actual rod and have a different texture. Multiple filaments may be present all at once (Fig. 8D, filaments 1–5). The padded end of filament D1 touches a *V. alginolyticus* rod. The padded ends of filaments D2 and D3 adhere to the glass substratum. More filaments are observed to directly attach to bacterial rods (Fig. 8E). Filaments E1 and E2 are making connections either on or directly next to a dividing *V. alginolyticus* rod.

These SSB01 filaments are also present when interacting with *T. mobilis* (Fig. 3C). The leaf-shaped pads at the ends of two filaments (C1 and C2) attach laterally to either side of a single *T. mobilis* rod. In general, all filaments do not appear to originate from any specific cell-surface structures of SSB01. There are no observations of SSE01 or SSA03 producing similar extracellular structures. These filaments do not appear to be described in any previous studies, and are distinctive from other structures, such as bacterial flagella, one of which can be seen in Fig. 8B (pink arrow). The much slender appearance of these filaments and their origination in multiples from the algal cell also distinguish them from the two flagella previously described by Trench and Blank [20], which we have also observed, connected by the peduncle (Fig. 8E, blue arrow).

### Extracellular material encase algae and facilitate interactions with bacteria

Across all strains of algae, both when inoculated with the beneficial *T. mobilis* or the pathogenic *V. alginolyticus*, an extracellular matrix (ECM) extends over and groups together multiple dinoflagellates and their immediate surrounding environment. Multiple SSB01 cells are encased together in such a matrix (Fig. 8F), in a wrinkled texture reminiscent of a biofilm and anchors the cell cluster to the glass substratum. ECM production also covers the substratum right above a trio of SSE01 cells (Fig. 4A and F, white arrows), embedding many different *T. mobilis* clusters within it. The ECM appears among SSA03 cells and *T. mobilis* clusters as well (Fig. 5D and F, white arrows). Though there is the possibility that this may be the result of exopolysaccharides produced by bacteria, this film appears in instances when bacterial cells are not making any direct contact with the algal surfaces.

### Genomic and physiological characterization of *Tritonibacter mobilis* AipH2 and *Vibrio alginolyticus* AipCC7 support metabolism of photosynthetic products

To determine the ability of *T. mobilis* AipH2 and *V. alginolyticus* AipCC7 to metabolize different carbon sources like glucose and glycerol, genomic metabolic analysis was performed with ModelSEED v2.6.1, which annotated the complete glucose metabolism pathway with key genes and intermediary products present for both bacterial strains (Fig. S4). For the metabolism of glycerol, *T. mobilis* AipH2 has glycerol-3-phosphate (G3P) (Table S1), a key intermediate and the first product of glycerol metabolism [56]. *V. alginolyticus* AipCC7, however, appears to have two more key intermediary compounds necessary in glycerol metabolism in addition to G3P. This includes the next compound in the pathway, dihydroxyacetone phosphate (DHAP), a dehydrogenated form of G3P [57]. DHAP isomerizes into glyceraldehyde-3-phosphate (GAP) [56], which is an important final step for entry into central metabolic pathways. Thus, *V. alginolyticus* AipCC7 may be more likely to use glycerol as a carbon source, corroborated by the results of substrate experiments described below.

To determine the responses of *T. mobilis* and *V. alginolyticus* to different algal products, we examined colony growth on M9 minimal media supplemented with various carbon sources pertinent to photosynthesis and/or host-microbe symbioses. Symbiodiniaceae produce both glucose and glycerol when they establish symbiosis with Aiptasia [54]. Their growth in culture is also enhanced with supplementation of amino acids in the form of casein hydrolysate [22]. Thus, we assessed whether these carbon sources are also useful to bacteria as we consider their interactions with Symbiodiniaceae. *V. alginolyticus* AipCC7 and *V. alginolyticus* AipCC7-GFP appear to be less selective than *T. mobilis* AipH2 and *T. mobilis* AipH2-GFP about their carbon source (Fig. S6), with capacity to grow on all compounds tested, likely due to more intermediate compounds being present in *V. alginolyticus* to facilitate glucose and glycerol metabolism.

## Discussion

Although the relationships of bacteria and dinoflagellates and their cnidarian hosts have been examined more closely in recent years, limited research has been done to look at the relationship between these two key microbes. More specifically, what facilitates cellular interactions between Symbiodiniaceae and bacteria in free-living or symbiotic forms remains unknown. In this study, we explored photosynthesis as a potential key factor in initiating physical contact between three Symbiodiniaceae species (*B. minutum, E. voratum*, and *S. pilosum*) and two bacterial species (one pathogenic and the other beneficial to corals and anemones).

### Role of photosynthesis in algal–bacterial interactions

Algal photosynthesis is important for establishing symbiosis with its cnidarian host [12]. Glucose, a product of photosynthesis, is a major metabolite transferred from endosymbiotic algae to its cnidarian host [50]. But the host may not be the only organism benefiting from this glucose supply. Glucose production from dinoflagellate photosynthesis may potentially influence microbial interactions as bacteria could be attracted to sugar production and respond to varying environmental glucose gradients through chemotaxis. *B. minutum* and *E. voratum* vary in levels of glucose and glycerol production when they colonize Aiptasia [54]. No research on the glucose production of *S. pilosum* has been published. Glucose and gluconic acid (a product of glucose usage in bacteria) were also recently identified metabolites exchanged between *B. minutum* and various bacterial genera [58]. Another key product of photosynthesis is molecular oxygen (O_2_). As algae photosynthesize, O_2_ is diffused into the surrounding environment. Positive aerotaxis, the attraction and navigation toward higher O_2_ gradients, is utilized by different species of marine bacteria to capitalize on this metabolic stockpile [59–61]. Both aerotactic and chemotactic responses (via photosynthesis-driven glucose gradients) may contribute toward how bacteria discover and interact with Symbiodiniaceae.

Our results indicate that localization of the beneficial bacterium, *T. mobilis*, to multiple species of Symbiodiniaceae is dependent on algal photosynthesis. Fluorescence-intensity measurements from GFP-tagged *T. mobilis* revealed a dramatic decrease in bacterial congregation around the single-celled dinoflagellates when photosynthesis was inhibited (Figs 1 and 2). Algae exposed to continuous darkness attracted significantly fewer bacteria, in contrast to those maintained on a regular diurnal cycle. While maintaining algae in the dark impacts the light-dependent reaction of photosynthesis, DCMU specifically inhibits electron flow from photosystem II to plastoquinone, halting the production of ATP and NADPH, and thereby impacting both the light-dependent and -independent (i.e. Calvin cycle) reactions of photosynthesis. Disruptions in algal–bacterial interactions might not simply be influenced by presence or absence of light alone, and hence, why we incorporated two methods to manipulate algal photosynthesis. Despite their different mechanisms in preventing photosynthesis, both DCMU (under a 12 h light:12 h dark cycle) and continuous darkness resulted in a decrease in bacterial congregation across all three algal strains, supporting the notion that photosynthesis is crucial for physical contact between *T. mobilis* and Symbiodiniaceae. Moreover, a genomic metabolic analysis of *T. mobilis* (Fig. S4, Table S1) corroborates its capacity to metabolize both glucose and glycerol that Symbiodiniaceae may be secreting. We do not yet know what mechanisms drive the physical contact between *T. mobilis* and Symbiodiniaceae in the absence of photosynthesis; however, our discovery sets up future questions to ask whether photosynthetic products such as glucose are initiating the observed interactions.

Our observations align with other studies that have suggested photosynthesis and its products to be an important factor in interactions between *Tritonibacter* spp. and photosynthetic microbes. When in co-culture with vitamin B12-depleted diatoms, *R. pomeroyi* experience an upregulation of relevant transmembrane proteins by 24- to 69-fold [35]*.* Such proteins include tripartite ATP-independent periplasmic transporter subunits, some of which are involved in the import of sugar derivatives into the bacterial cell*.* Similarly, significant increases in anemone-protein content were observed when Aiptasia was inoculated with *T. mobilis*, but only when algal endosymbionts were also present [34]. This suggests that these interactions may benefit not only the microbes involved but also the entire holobiont (the host and its associated microbes). New evidence [62] continues to support a beneficial *Tritonibacter-*Symbiodiniaceae relationship by identifying nitrogen exchange between the two microbes, followed by an increase in photosynthetic capacity and algal proliferation as a consequence. Given the other key product of photosynthesis is oxygen, possible aerotaxis of *Tritonibacter* towards Symbiodiniaceae is worthwhile to examine in future studies. These data combined continue to stress the importance of exploring what initiates these interactions. Although more combinations of Symbiodiniaceae and beneficial bacteria should be analyzed, along with concrete measurements of glucose and oxygen exchange (as products of photosynthesis), we have at least identified photosynthesis to be one major factor in this relationship establishment.

Among beneficial bacteria in the cnidarian holobiont, others such as *Labrenzia alexandrii*, *Marinobacter adhaerens*, and another *Tritonibacter* sp*.* have been shown to increase Symbiodiniaceae proliferation through different mechanisms [58, 62] and rescue photosynthetic health under heat stress [63]. The photosynthetic link between *T. mobilis* and Symbiodiniaceae suggests that similar molecular mechanisms may be shared among different species of beneficial bacteria in their interactions with dinoflagellates*.* Additionally, SEM imaging from this study reveals instances in which individual *T. mobilis* cells attach to SSB01 algae mainly by the polar ends of the rod (Fig. 3), where specialized protein–protein interactions possibly take place. *T. mobilis* attachment to SSE01 and SSA03 algae in varying orientations (Figs 4 and 5) suggests a possible diversity of pattern recognition receptors involved in interactions between the two microbes. These may subsequently induce signal cascades involved in the beneficial roles of *Tritonibacter*. More SEM analysis should be conducted to determine whether such physical interactions are conserved between different beneficial bacteria and Symbiodiniaceae.

### Alternative mechanisms employed by *Vibrio* in interacting with algae

Microscopic analysis of a common pathogen to cnidarians revealed striking differences in its interactions with Symbiodiniaceae. In contrast to *T. mobilis, V. alginolyticus* adheres to dinoflagellates regardless of photosynthetic inhibition (Figs 6 and 7). SEM analysis revealed adhesion of this pathogen to the algal surface to be random and indiscriminate of any key landmarks such as the large dimple or divots produced by the wrinkled texture of the algal-cell surface (Fig. 8). These results are consistent with how *V. alginolyticus* grows in culture on its own, as it is also indiscriminate in its temperature and substrate preferences, unlike *T. mobilis* that is more selective (Figs S5 and S6). Our analysis of bacterial fluorescence intensity showed a consistent congregation of *V. alginolyticus* around the algal cells across all three strains and all photosynthetic treatments. A wide range of studies have reported numerous pathogenic *Vibrio* spp. targeting non-photosynthetic microbes and tissues indiscriminately [43, 45, 46, 64-67]. For example, *V. shiloi* physically adheres to the algal endosymbiont as well as the cnidarian tissue in infected coral samples [45] with no specific distinction between the two. However, it was later observed [68] that DCMU-treated corals do not secrete a receptor in their mucus for *V. shiloi* recognition. Hence, even though our study suggests photosynthesis is not a driver in direct *Vibrio*-Symbiodiniaceae recognition, photosynthesis might still be involved indirectly; further research into the recognition process within the host is necessary. More investigation of *Vibrio*-Symbiodiniaceae recognition is vital to our understanding of host health and immunity, and specific pattern recognition receptors and microbial-associated molecular patterns involved should be explored.

Bacterial pathogens have evolved to produce variable secretion systems and use diverse mechanisms to successfully infiltrate their target. *V. alginolyticus* is known to utilize a wide variety of infection mechanisms such as a type III secretion system, type VI secretion system, quorum sensing, extracellular proteases, motility, siderophore-iron-dependent uptake systems, biofilm formation, and adhesion [67, 69]. One crucial virulence factor is adhesion, or the ability of the pathogen to recognize and attach to its host. Many *Vibrio* spp. utilize an outer membrane protein known as OmpU for host recognition and virulence, binding the *Vibrio* pathogen to β-integrin and inducing clathrin-mediated endocytosis in the animal-host cell [70, 71]. Such a protein is a viable candidate to explore how *V. alginolyticus* specifically binds to Symbiodiniaceae.

### Putative molecular mechanisms underlying physical contact between Symbiodiniaceae and bacteria

Protein–protein interactions must be researched to fully understand the signaling pathways utilized between algae and bacteria. Beneficial interactions observed between other photosynthetic organisms and *Tritonibacter* spp. (or other Roseobacters) may shed light on possible protein–protein interactions that occur within our system. Co-culturing of a diatom and *Tritonibacter* sp*.* yielded upregulation of different leucine-rich repeat (LRR) receptor genes in the diatom [35]. LRR receptors are known to be used in photosynthetic cells for recognizing bacterial proteins like flagellin, inducing a signaling cascade that activates MAPK and calcium-dependent protein kinase pathways connected to immune defense and development [72–76]. Moreover, co-culturing with *R. pomeroyi* induced upregulation of C-type lectins and other bacterial-recognition genes in the green alga *Micromonas commoda* [77]. C-type lectins are another type of transmembrane protein used for recognizing specific glycans on other cell surfaces, including those of bacteria [78]. The lectin-glycan mechanism appears to be prominent in establishing initial attachment of Roseobacters to their hosts. Polar glycans of the bacterium *Phaeobacter inhibens* are involved in its attachment to the coccolithophore *Emiliania huxleyi* [79]. In terrestrial models, a specific glycan solely localized to the polar end of rhizobia bacteria enables their binding to lectins of their host plant [80]. Symbiodiniaceae, such as *B. minutum*, do contain many different LRR receptors, C-type lectins, and other transmembrane proteins in general [81] that could be involved in possible signal transduction when in contact with diverse bacteria*.*

### Future directions

Electron-microscopy analysis revealed the presence of unknown filaments extending from *B. minutum*. More analysis on these morphological features should be done to investigate their purpose, functions, and conditions required to induce filament formation. Transcriptomics of all Symbiodiniaceae and bacterial combinations under both normal and photosynthesis-inhibition conditions may validate the candidate genes mediating photosynthesis-dependent interactions.

Photosynthetic and non-photosynthetic organisms continue to be impacted on an ecological level that can potentially affect microbial interactions on a cellular level. One increasingly prevalent anthropogenic factor is heat stress induced by climate change. Rising ocean temperatures not only induce bleaching in cnidarians but also in Symbiodiniaceae [11], severely reducing the photochemical output of photosystem II, depleting chlorophyll pigmentation, and weakening the cell [9, 10]. This will impact both free-living and symbiotic microbes. Such stress exacerbates the vulnerability of the entire holobiont via amplifying the virulence of pathogens [69] and disruption of both dinoflagellate [82, 83] and cnidarian microbiomes [28]. Microscopic analysis of heat-stressed dinoflagellates and their interactions with bacteria should be further studied to uncover the consequences of climate change on the microbial level.

In conclusion, we evaluated the relevance of photosynthesis in initiating physical interactions between algae and bacteria. *T. mobilis*, a beneficial bacterium to corals, is attracted to photosynthetic Symbiodiniaceae, but not when photosynthesis is inhibited. *V. alginolyticus*, a coral pathogen, adheres to algae indiscriminately, independent of photosynthesis or its suppression. Thus, algal photosynthesis appears to be an important factor in establishing contact with some bacteria, but it is certainly not the only factor. Many combinations between beneficial bacteria and Symbiodiniaceae must be analyzed to validate the possible conservation of this photosynthetic link between microbes. Furthermore, these investigations need to be conducted *in hospite* in corals and other cnidarian models (e.g. Aiptasia) as well. As a result, our findings pave the way for continuing to explore photosynthesis and other potential mechanisms that mediate microbe-microbe interactions among free-living species and those within the cnidarian holobiont.

## Supplementary Material

McLaren_et_al_Supplementary_Material_250303_ycaf070

## Data Availability

The datasets presented in this study are available in online repositories. The genome sequences of *Tritonibacter mobilis* AipH2 and *Vibrio alginolyticus* AipCC7 have been deposited at the NCBI Genome database under the accession numbers SAMN45194499 and SAMN45194500, respectively, associated with BioProject PRJNA716944.
