# Supplementary material for: Photosynthetic dependence and filament production in physical bacterial–Symbiodiniaceae interactions
Source: ISME Commun. 2025 Apr 25;5(1):ycaf070. doi: 10.1093/ismeco/ycaf070 (PMC12082827; doi:10.1093/ismeco/ycaf070)
Supplement: McLaren_et_al_Supplementary_Material_250303_ycaf070 [file mclaren_et_al_supplementary_material_250303_ycaf070.pdf]

Supplementary Materials and Methods

Sequencing of bacterial genomes

To sequence the genomes of *Tritonibacter mobilis* AipH2 and *Vibrio alginolyticus* AipCC7, genomic DNA was extracted using a Quick-DNA Fungal/Bacteria Miniprep Kit (Zymo Research, Irvine, CA, USA). DNA was submitted to the Plasmidsaurus Sequencing Center (Eugene, OR, USA) for library preparation (Oxford Nanopore Rapid Sequencing Kit V14) and whole-genome sequencing (Oxford Nanopore Sequencing, R10.4.1 flow cell), producing 113,741 total reads for *T. mobilis* AipH2, and 257,687 total reads for *V. alginolyticus* AipCC7. Reads were trimmed using Fitlong v0.2.1 [1] assembled with Flye v2.9.3 [2] and polished with Racon v1.4.20 [3] to determine if any contigs were circular by identifying overlapping regions. Genome annotation was conducted with Prokka v1.14.5 [4] and the NCBI Prokaryotic Genome Annotation Pipeline (PGAP) v5.1 [5]. *T. mobilis* AipH2 has a 4.99-Mb genome, a total GC content of 58.94% with six contigs at 155x coverage, and a contig N_{50} value of 3.1-Mb, with 4,739 predicted coding sequences. *V. alginolyticus* AipCC7 has a 5.24-Mb genome, a total GC content of 44.67% with three contigs at 291x coverage, and a contig N_{50} value of 2.7-Mb, with 4,760 predicted coding sequences. Default parameters were used except when noted. Phylogenies were constructed via the codon tree method with PATRIC and RAxML alignment [6–8], using 100-single copy genes and proteins annotated with PGFams [9, 10]. Phylogenic trees were visualized in FigTree (v1.4.4; [11]).

Phenotypic validation of GFP-tagged bacterial strains in response to different temperatures

To determine temperature preferences of *T. mobilis* AipH2, *T. mobilis* AipH2-GFP, *V. alginolyticus* AipCC7, and *V. alginolyticus* AipCC7-GFP, all four strains were grown on marine agar and in triplicate cultures of marine broth (MB), then monitored at three different temperatures. An ambient temperature of 27°C is the common temperature used to maintain the clonal lines of Aiptasia [12] in which the bacteria were originally isolated from. A higher temperature of 34°C is often used in heat-stress studies that result in cnidarian bleaching [13, 14] and changes to the microbiome [12]. A much higher temperature of 42°C in the present study tests the thermophilic properties (if any) of all four bacterial strains of interest. Marine-agar plates with all strains were incubated at those temperatures for 24 h to assess growth of colonies for each strain. Growth rates of MB cultures, incubating at those temperatures and shaking at 200 rpm, were compared via cell-density measurements (OD_{600}) using a Thermofisher Scientific Spectronic 200E.

Supplementary Results

Classification of bacterial strains

Phylogenetic analyses confirmed that strain *T. mobilis* AipH2 belongs to the genus *Tritonibacter* (formerly *Ruegeria* [15], Fig. S2), the family Paracoccacceae (formerly Rhodobacteraceae) [16, 17] and the class Alphaproteobacteria. The Average Nucleotide Identity (ANI, CJ Bioscience) value of *T. mobilis* AipH2 and its next closely related species *R. mobilis* strain NBRC101030 was 96.76% [4, 18]. *V. alginolyticus* AipCC7 belongs to the genus *Vibrio* (Fig. S3), the family Vibrionaceae and the class Gammaproteobacteria [19]. Comparing *V.*

alginolyticus AipCC7 with *V. alginolyticus* E110, the average ANI was 98.65%. The strains isolated from Aiptasia have high percent similarity (> 95%) to their closely related species, designating them as strains.

Growth dynamics are similar between wildtype and GFP-tagged bacterial strains

Wildtype and GFP-labeled strains of *T. mobilis* and *V. alginolyticus* were compared to assess any impacts of GFP insertion on bacterial growth dynamics. Bacterial densities, as determined by OD_{600} values, varied across 27°C (ambient temperature), 34°C (temperature inducing heat stress in cnidarians), and 42°C. At both 27°C and 34°C, *T. mobilis* AipH2 and *T. mobilis* AipH2-GFP grew at similar rates (Fig. S5, A-B), but neither could grow at 42°C (Fig. S5, C). In contrast, *V. alginolyticus* AipCC7 and *V. alginolyticus* AipCC7-GFP tolerated high temperatures better and grew at 42°C at rates comparable to those at 27°C and 34°C. Growth of *V. alginolyticus* AipCC7-GFP was slightly slower than *V. alginolyticus* AipCC7 at all temperatures, but both wildtype and fluorescent strains still grew faster than their *T. mobilis* counterparts overall. Growth on marine agar also confirmed these same temperature preferences across all strains (Fig. S5, D-F). These results show that *T. mobilis* AipH2 and *V. alginolyticus* AipCC7 have vastly different temperature preferences when compared to each other, but GFP insertion had no major impact on the growth dynamics of each species.

Supplementary Table and Figures

Table S1. Compounds involved in glucose and glycerol metabolism of *Tritonibacter mobilis* AipH2 and *Vibrio alginolyticus* AipCC7. ModelSEED analysis of *T. mobilis* AipH2 and *V. alginolyticus* AipCC7 genomes revealed key compounds involved in glucose and glycerol metabolism. *V. alginolyticus* AipCC7 appears to be better suited to metabolize glycerol, as it produces more intermediary compounds for this process.

Bacterium	Function	Name	Formula	Charge
Tritonibacter mobilis AipH2	Glycerol Metabolism	Glycerol-3-phosphate	C3H7O6P	-2
Tritonibacter mobilis AipH2	Glucose Metabolism	UDP-glucose	C15H22N2O17P2	-2
Tritonibacter mobilis AipH2	Glucose Metabolism	Glucose-1-phosphate	C6H11O9P	-2
Tritonibacter mobilis AipH2	Glucose Metabolism	beta-D-Glucose	C6H12O6	0
Tritonibacter mobilis AipH2	Glucose Metabolism	beta-D-Glucose 6-phosphate	C6H11O9P	-2
Tritonibacter mobilis AipH2	Glucose Metabolism	alpha-D-Glucose	C6H12O6	0
Tritonibacter mobilis AipH2	Glucose Metabolism	alpha-D-Glucose 6-phosphate	C6H11O9P	-2
Vibrio alginolyticus AipCC7	Glycerol Metabolism	Glycerol-3-phosphate	C3H7O6P	-2
Vibrio alginolyticus AipCC7	Glycerol Metabolism	Glycerone-phosphate (dihyroxyacetone phosphate DHAP)	C3H5O6P	-2
Vibrio alginolyticus AipCC7	Glycerol Metabolism	Glyceraldehyde3-phosphate	C3H5O6P	-2
Vibrio alginolyticus AipCC7	Glycerol Metabolism	3-Phosphoglycerate	C3H4O7P	-3
Vibrio alginolyticus AipCC7	Glycerol Metabolism	1,3-Bisphospho-D-glycerate	C3H4O10P2	-4
Vibrio alginolyticus AipCC7	Glycerol Metabolism	Glycerate	C3H5O4	-1
Vibrio alginolyticus AipCC7	Glycerol Metabolism	2-Phospho-D-glycerate	C3H4O7P	-3
Vibrio alginolyticus AipCC7	Glucose Metabolism	UDP-glucose	C15H22N2O17P2	-2
Vibrio alginolyticus AipCC7	Glucose Metabolism	Glucose-1-phosphate	C6H11O9P	-2
Vibrio alginolyticus AipCC7	Glucose Metabolism	UDPglucuronate	C15H19N2O18P2	-3
Vibrio alginolyticus AipCC7	Glucose Metabolism	beta-D-Glucose	C6H12O6	0
Vibrio alginolyticus AipCC7	Glucose Metabolism	6-Phospho-D-gluconate	C6H10O10P	-3
Vibrio alginolyticus AipCC7	Glucose Metabolism	beta-D-Glucose 6-phosphate	C6H11O9P	-2
Vibrio alginolyticus AipCC7	Glucose Metabolism	1-Phospho-alpha-D-glucuronate	C6H8O10P	-3
Vibrio alginolyticus AipCC7	Glucose Metabolism	6-phospho-D-glucono-1-5-lactone	C6H9O9P	-2

Vibrio alginolyticus AipCC7	Glucose Metabolism	N-Glucosylnicotinate	$C_{12}H_{15}NO_7$	0
Vibrio alginolyticus AipCC7	Glucose Metabolism	5'-O-beta-D-Glucosylpyridoxine	$C_{14}H_{21}NO_8$	0
Vibrio alginolyticus AipCC7	Glucose Metabolism	beta-D-Glucuronic acid	$C_6H_9O_7$	-1
Vibrio alginolyticus AipCC7	Glucose Metabolism	alpha-D-Glucose	$C_6H_{12}O_6$	0
Vibrio alginolyticus AipCC7	Glucose Metabolism	alpha-D-Glucose 6-phosphate	$C_6H_{11}O_9P$	-2

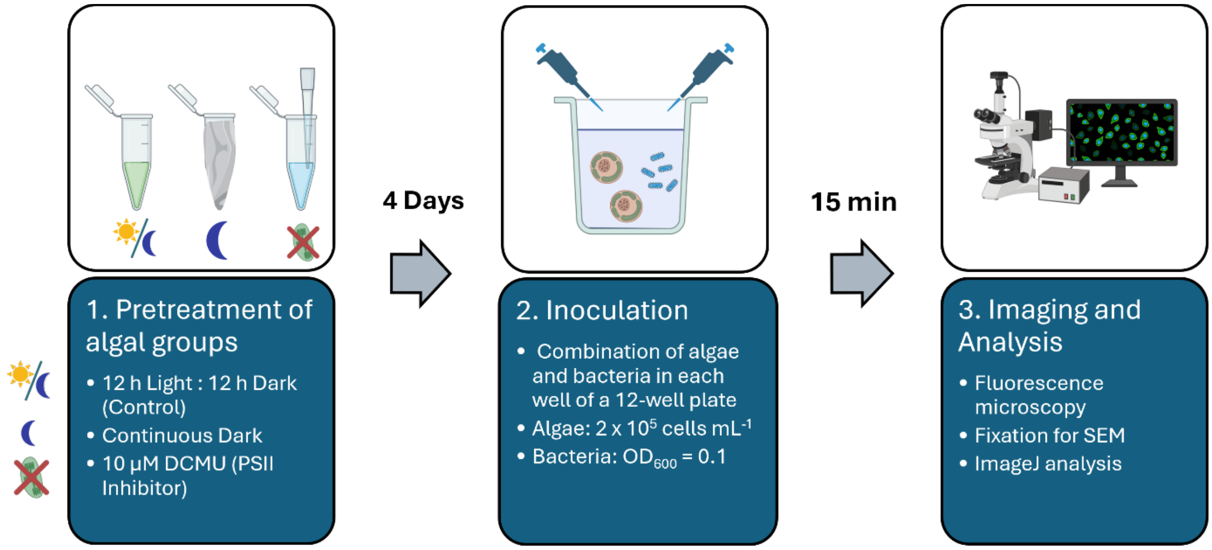

Figure S1. Experimental workflow in assessing the role of photosynthesis in dinoflagellate-bacterial interactions. Dinoflagellates of the Symbiodiniaceae family were pre-treated in the following conditions for 4 days: (1) 12 h light : 12 h dark (control), (2) continuous dark, and (3) 10 µM DCMU (photosystem II inhibitor). Cell densities were standardized for both dinoflagellates and bacteria prior to combining. Live cells and their physical interactions were observed after ~15 min under fluorescence microscopy or fixed and preserved for scanning electron microscopy (see Materials and Methods). Images were subsequently analyzed with Image J (version 1.54f). Figure created with biorender.com.

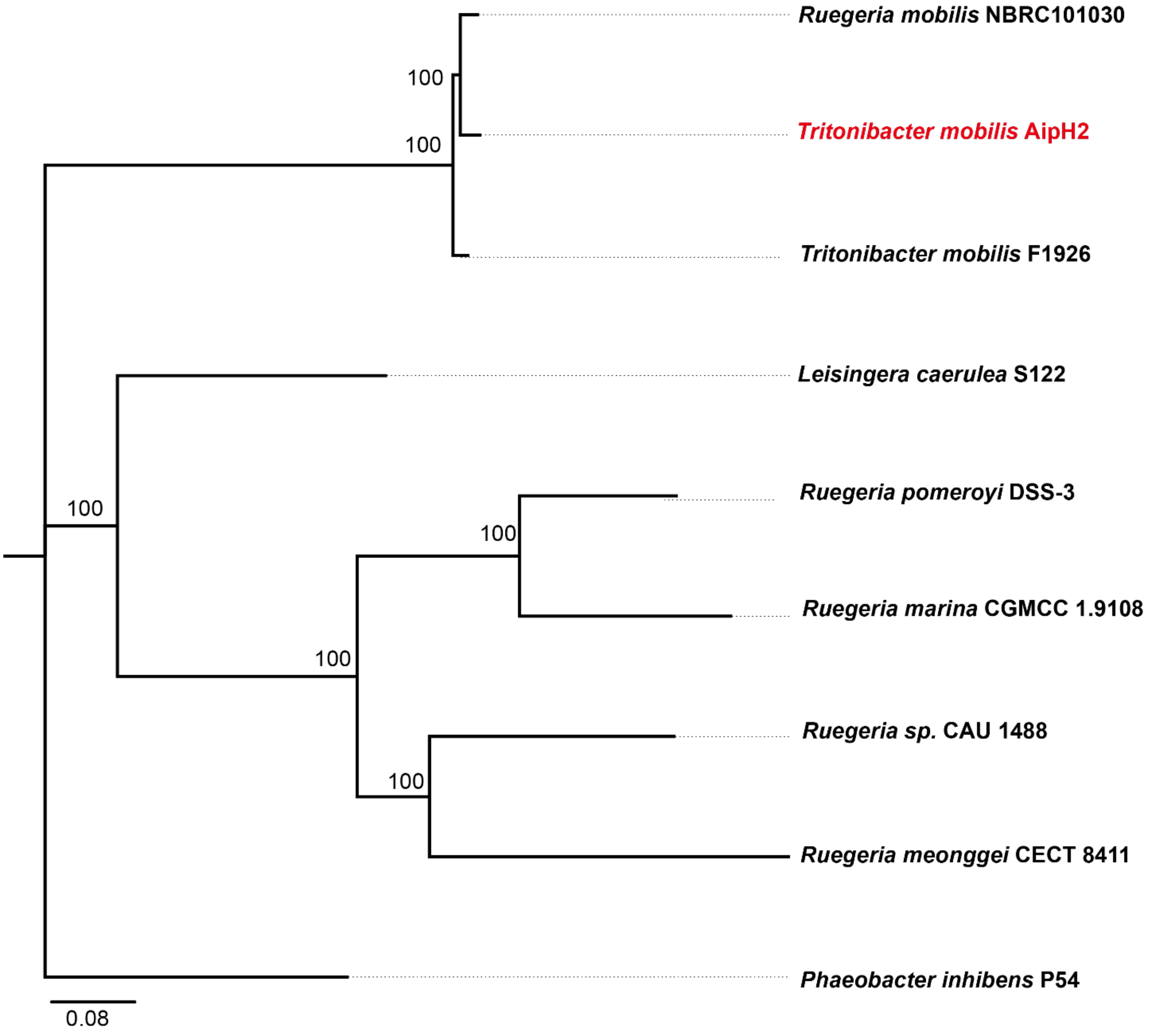

Figure S2. Phylogeny of bacterium *Tritonibacter mobilis* strain AipH2. A maximum likelihood phylogeny of *Tritonibacter mobilis* AipH2 (red) constructed via the codon tree method through PATRIC, using 100 single-copy genes and proteins identified by PGFams. GenBank Accession numbers for the phylogenetic tree are as follows: *Ruegeria mobilis* strain NBRC101030 (LNWY00000000), *Tritonibacter mobilis* F1926 (AQCH00000000), *Leisingera caerulea* S122 (CP081070), *Ruegeria pomeroyi* DSS-3 (CP000031), *Ruegeria marina* strain CGMCC 1.9108 (FMZV00000000), *Ruegeria* sp. CAU 1488 (VCPD00000000), *Ruegeria meonggei* strain CECT 8411 (FWFP00000000), and *Phaeobacter inhibens* strain P54 (CP010650).

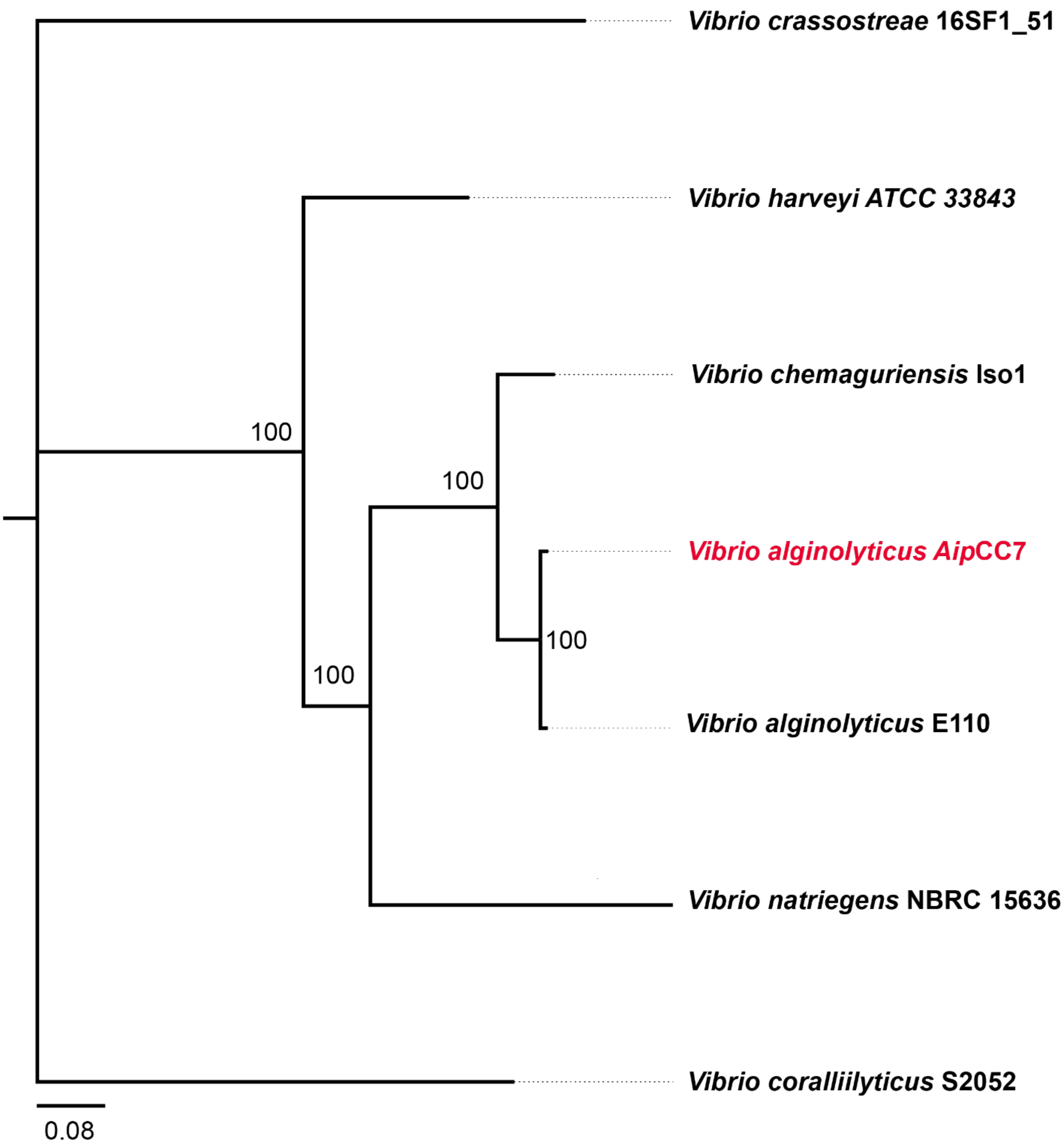

Figure S3. Phylogeny of bacterium *Vibrio alginolyticus* strain AipCC7. A maximum likelihood phylogeny of *Vibrio alginolyticus* AipCC7 (red) constructed via the codon tree method through PATRIC, using 100 single-copy genes and proteins identified by PGFams. GenBank Accession numbers for the phylogenetic tree are as follows: *Vibrio crassostreae* strain 16SF1_51 (RJKJ00000000), *Vibrio harveyi* strain ATCC (CP009467), *Vibrio chemaguriensis* strain Iso1 (SHOE00000000), *Vibrio alginolyticus* E110 (CP098033), *Vibrio natriegens* NBRC 15636 (CP009977), and *Vibrio coralliilyticus* S2052 (JXXR00000000).

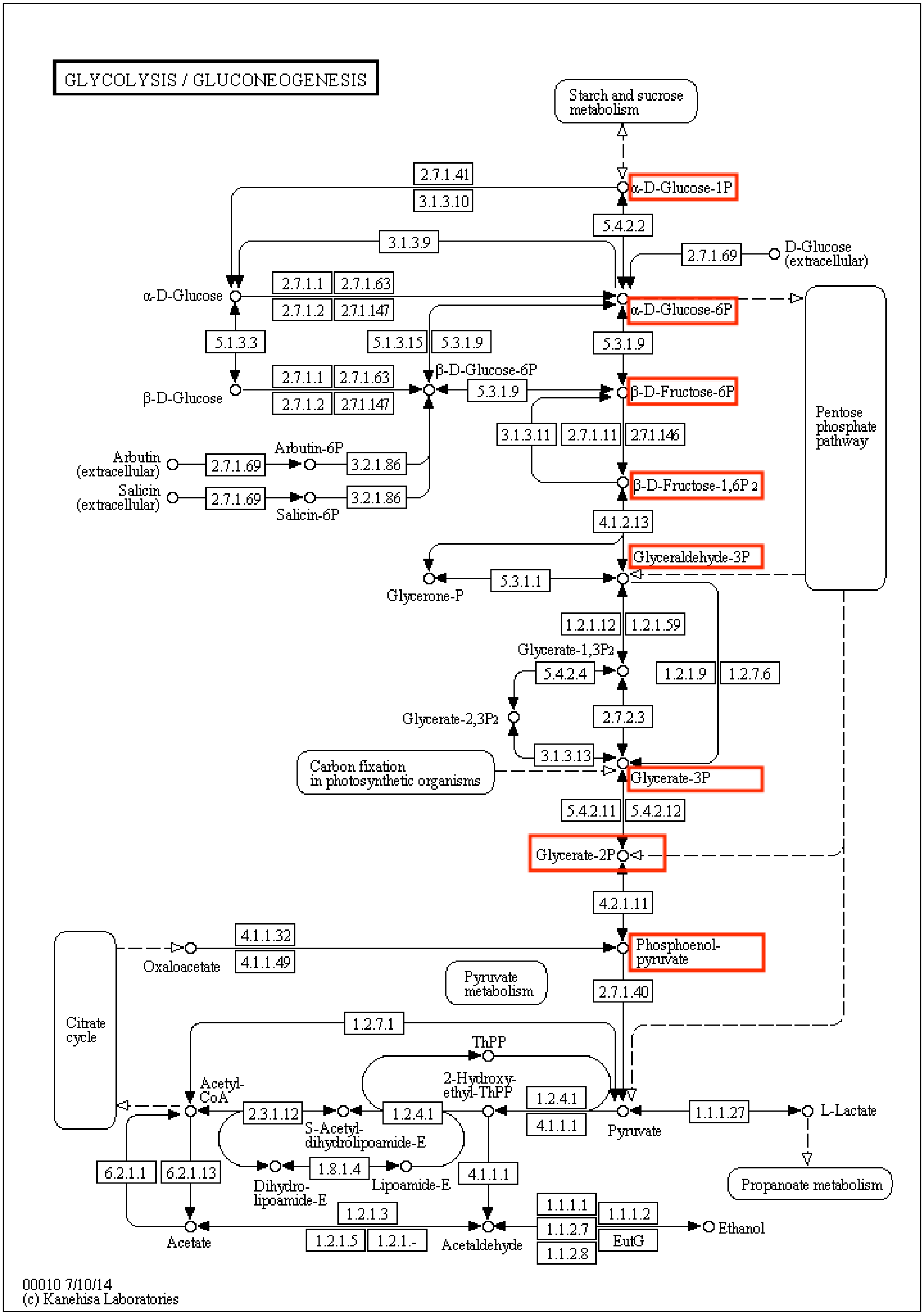
GLYCOLYSIS / GLUCONEOGENESIS
Starch and sucrose metabolism
α-D-Glucose-1P
2.7.1.41
3.1.3.10
5.4.2.2
3.1.3.9
2.7.1.69
D-Glucose (extracellular)
α-D-Glucose
2.7.1.1
2.7.1.63
2.7.1.2
2.7.1.147
α-D-Glucose-6P
5.1.3.3
5.1.3.15
5.3.1.9
β-D-Glucose-6P
β-D-Glucose
5.3.1.9
β-D-Fructose-6P
Pentose phosphate pathway
3.1.3.11
2.7.1.11
2.7.1.146
Arbutin (extracellular)
Arbutin-6P
3.2.1.86
Salicin (extracellular)
Salicin-6P
β-D-Fructose-1,6P2
4.1.2.13
Glyceraldehyde-3P
5.3.1.1
Glycerone-P
1.2.1.12
1.2.1.59
Glycerate-1,3P2
5.4.2.4
1.2.1.9
1.2.7.6
Glycerate-2,3P2
2.7.2.3
3.1.3.13
Carbon fixation in photosynthetic organisms
Glycerate-3P
5.4.2.11
5.4.2.12
Glycerate-2P
4.2.1.11
Citrate cycle
Oxaloacetate
4.1.1.32
4.1.1.49
Phosphoenol-pyruvate
Pyruvate metabolism
2.7.1.40
1.2.7.1
ThPP
2-Hydroxy-ethyl-ThPP
Acetyl-CoA
2.3.1.12
1.2.4.1
4.1.1.1
Pyruvate
1.1.1.27
L-Lactate
S-Acetyl-dihydrolipoamide-E
1.8.1.4
Dihydro-lipoamide-E
Lipoamide-E
6.2.1.1
6.2.1.13
Propanoate metabolism
1.2.1.3
1.1.1.1
1.1.1.2
1.1.2.7
EutG
1.1.2.8
Ethanol
Acetate
1.2.1.5
1.2.1.-
Acetaldehyde
00010 7/10/14
(c) Kanehisa Laboratories

Figure S4. Metabolic model of glucose metabolism by *Tritonibacter mobilis* AipH2 and *Vibrio alginolyticus* AipCC7. ModelSEED v2.6.1 analysis was performed on bacterial strains *T. mobilis* AipH2 and *V. alginolyticus* AipCC7 which found that both strains have complete glycolysis pathways to metabolize glucose. Image was generated from ModelSEED pathway analysis. Key products in the glucose metabolism process are highlighted in red boxes.

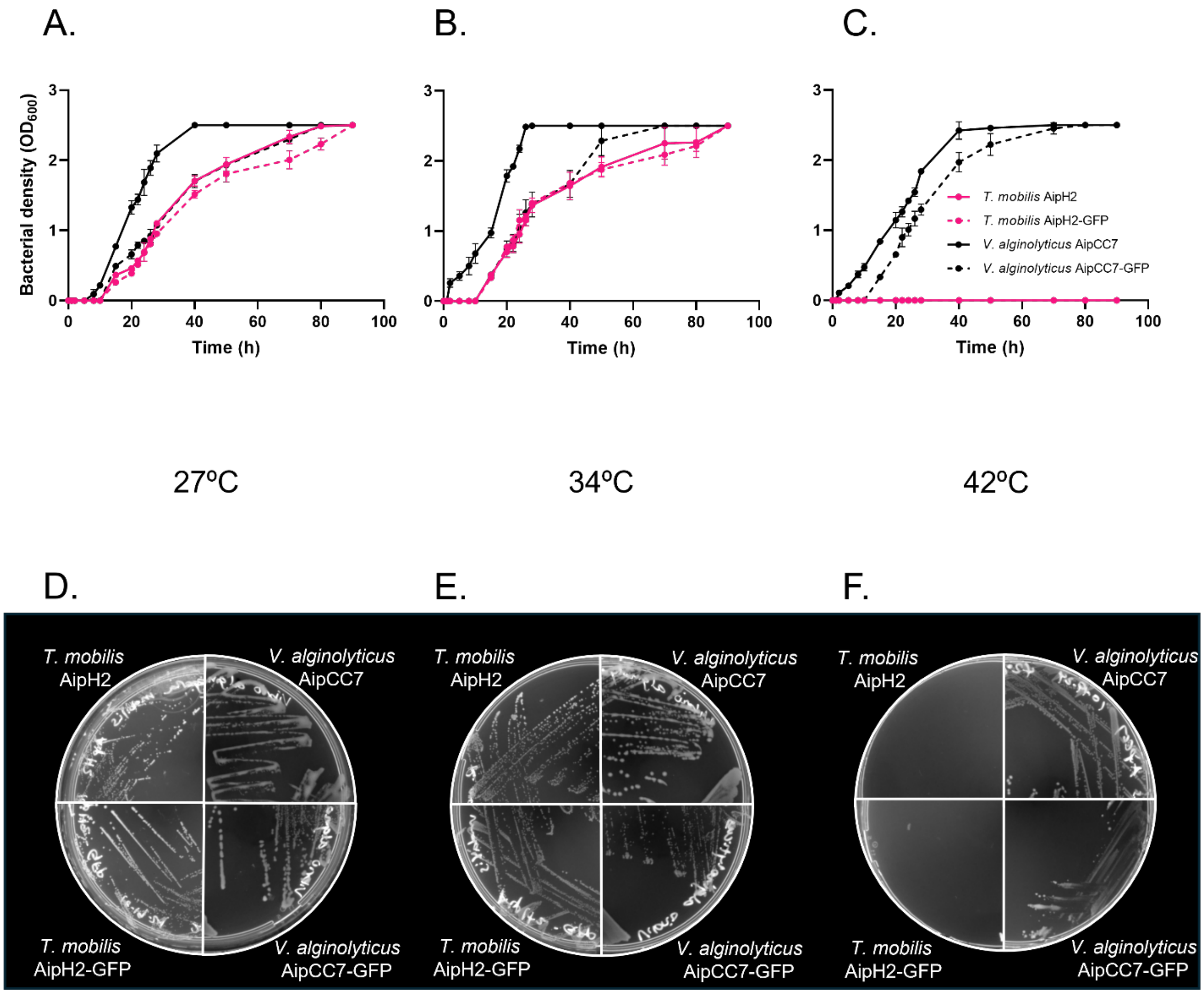

Figure S5. Growth of wildtype and GFP-tagged strains of *Tritonibacter mobilis* and *Vibrio alginolyticus* at different temperatures. Bacterial densities (OD_{600}) of *T. mobilis* AipH2, *T. mobilis* AipH2-GFP, *V. alginolyticus* AipCC7, and *V. alginolyticus* AipCC7-GFP grown in marine broth were monitored at (A) 27°C, (B) 34°C, and (C) 42°C until cultures reached stationary phase. Points represent average bacterial intensity ± SD across three replicates of each strain. Growth of each strain on marine agar was also assessed at (D) 27°C, (E) 34°C, and (F) 42°C after incubation for 24 h.

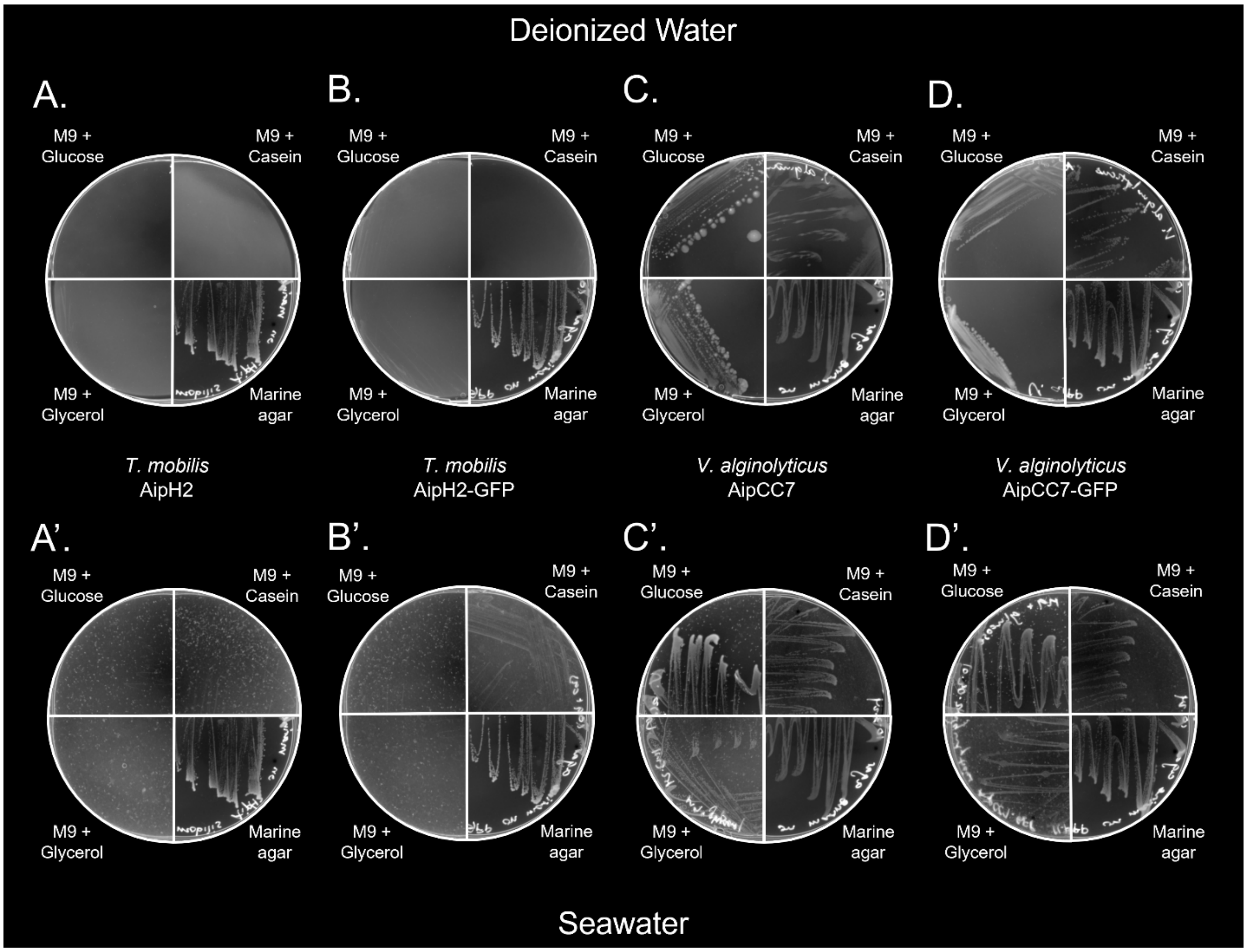

Figure S6. Substrate preferences of wildtype and GFP-tagged strains of *Tritonibacter mobilis* and *Vibrio alginolyticus*. Growth of *T. mobilis* AipH2, *T. mobilis* AipH2-GFP, *V. alginolyticus* AipCC7, and *V. alginolyticus* AipCC7-GFP on marine agar and solid M9 minimal media made in deionized water (A-D) and seawater (A'-D'), supplemented with 0.4% glucose, 0.4% glycerol, or 0.4% casein hydrolysate as carbon sources. All agar plates were incubated at 27°C for 24 h in the cases of marine agar and M9 in seawater, or 72 h in the case of M9 in deionized water (in which bacteria took longer to grow).

Supplementary References

1. Wick R, Menzel P. Filtlong. 2017. github. https://github.com/rrwick/Filtlong (11 November 2024, date last accessed).
2. Kolmogorov M et al. metaFlye: scalable long-read metagenome assembly using repeat graphs. *Nat Methods* 2020;**17**:1103–1110. https://doi.org/10.1038/s41592-020-00971-x
3. Vaser R et al. Fast and accurate de novo genome assembly from long uncorrected reads. *Genome Res* 2017;**27**:737–746. https://doi.org/10.1101/gr.214270.116
4. Seemann T. Prokka: rapid prokaryotic genome annotation. *Bioinformatics* 2014;**30**:2068–2069. https://doi.org/10.1093/bioinformatics/btu153
5. Tatusova T et al. NCBI prokaryotic genome annotation pipeline. *Nucleic Acids Res* 2016;**44**:6614–6624. https://doi.org/10.1093/nar/gkw569
6. Olson RD et al. Introducing the Bacterial and Viral Bioinformatics Resource Center (BV-BRC): a resource combining PATRIC, IRD and ViPR. *Nucleic Acids Research* 2023;**51**:D678–D689. https://doi.org/10.1093/nar/gkac1003
7. Davis JJ et al. PATtyFams: Protein families for the microbial genomes in the PATRIC Database. *Front Microbiol* 2016;**7**. https://doi.org/10.3389/fmicb.2016.00118
8. Stamatakis A. RAxML version 8: a tool for phylogenetic analysis and post-analysis of large phylogenies. *Bioinformatics* 2014;**30**:1312–1313. https://doi.org/10.1093/bioinformatics/btu033
9. Letunic I, Bork P. Interactive tree of life (iTOL) v3: an online tool for the display and annotation of phylogenetic and other trees. *Nucleic Acids Res* 2016;**44**:W242–W245. https://doi.org/10.1093/nar/gkw290

10. Price MN, Dehal PS, Arkin AP. FastTree 2 – Approximately maximum-likelihood trees for large alignments. *PLoS ONE* 2010;**5**:e9490. https://doi.org/10.1371/journal.pone.0009490

11. Rambaut A. FigTree v1. 3.1 Institute of Evolutionary Biology, University of Edinburgh, Edinburgh. 2010. 2010.

12. Sydnor JR et al. Changes in the microbiome of the sea anemone *Exaiptasia diaphana* during bleaching from short-term thermal elevation. *Front Mar Sci* 2023;**10**:1130964. https://doi.org/10.3389/fmars.2023.1130964

13. Bieri T et al. Relative contributions of various cellular mechanisms to loss of algae during cnidarian bleaching. *PLoS ONE* 2016;**11**:e0152693. https://doi.org/10.1371/journal.pone.0152693

14. Cleves PA et al. Insights into coral bleaching under heat stress from analysis of gene expression in a sea anemone model system. *Proc Natl Acad Sci USA* 2020;**117**:28906–28917. https://doi.org/10.1073/pnas.2015737117

15. Wirth JS, Whitman WB. Phylogenomic analyses of a clade within the Roseobacter group suggest taxonomic reassignments of species of the genera *Aestuariivita, Citreicella, Loktanella, Nautella, Pelagibaca, Ruegeria, Thalassobius, Thiobacimonas* and *Tropicibacter*, and the proposal of six novel genera. *International Journal of Systematic and Evolutionary Microbiology* 2018;**68**:2393–2411. https://doi.org/10.1099/ijsem.0.002833

16. Göker M. Filling the gaps: missing taxon names at the ranks of class, order and family. *International Journal of Systematic and Evolutionary Microbiology* 2022;**72**. https://doi.org/10.1099/ijsem.0.005638

17. Liang KYH et al. Roseobacters in a sea of poly- and paraphyly: Whole genome-based taxonomy of the family Rhodobacteraceae and the proposal for the split of the

“Roseobacter Clade” Into a novel family, Roseobacteraceae fam. nov. *Front Microbiol* 2021;**12**:683109. https://doi.org/10.3389/fmicb.2021.683109

18. Yoon S-H et al. Introducing EzBioCloud: a taxonomically united database of 16S rRNA gene sequences and whole-genome assemblies. *International Journal of Systematic and Evolutionary Microbiology* 2017;**67**:1613–1617. https://doi.org/10.1099/ijsem.0.001755

19. Miyamoto Y, Nakamuma K, Takizawa K. Pathogenic halophiles. Proposals of a new genus ‘Oceanomonas’ and of the amended species names. *Japanese Journal of Microbiology* 1961;**5**:477–486.
